# Trends and Factors Associated with Oral Contraceptive Use among Korean Women

**DOI:** 10.3390/healthcare9101386

**Published:** 2021-10-16

**Authors:** Hyejin Park, Kisok Kim

**Affiliations:** 1 Department of International Healthcare Administration, Daegu Catholic University, Gyeongsan 38430, Korea; hjpark@cu.ac.kr; 2 College of Pharmacy, Keimyung University, Daegu 42601, Korea

**Keywords:** oral contraceptive, annual trends, sociodemographic factors, postmenopausal women, KNHANES

## Abstract

Although oral contraceptives (OC) are widely used, few national-level epidemiologic studies have evaluated the prevalence of OC use and factors related to their use in Korea. We performed a population-based cross-sectional study on OC use by premenopausal women aged 20–59 years residing in Korea. We used secondary data from the 2010–2019 National Health and Nutrition Examination Survey to examine trends in the annual prevalence of OC use between 2010 and 2019, and factors influencing OC use. Based on data from 14,386 premenopausal women, the average annual prevalence of OC use was 8.2–10.7% between 2010 and 2014; it increased to 12.6–14.4% during 2015–2019. The prevalence of OC use was significantly higher in women with higher (≥5) than lower gravidity (<5). In addition, among sociodemographic factors, education level, household income, cigarette smoking, and alcohol drinking were significantly associated with OC use in Korean women. As OC use is affected by sociodemographic factors, a contraceptive plan that considers sociodemographic factors is needed to establish an effective family planning policy.

## 1. Introduction

Advances in contraceptive methods are an important achievement in the field of public health, with about 60% of women of childbearing age using modern methods of contraception [1]. Oral contraceptives (OCs) are an important and widely accepted method of contraception worldwide [2]. OCs are used by more than 150 million women worldwide, as an effective and reversible method of family planning [3,4]. In general, OCs are among the most commonly used methods of contraception in developed countries. However, the prevalence of OC use is lower in developed Asian countries, such as Japan and Korea, than in Western countries [5], suggesting differences in the sociodemographic factors affecting the prevalence of OC use.

Understanding the factors influencing contraceptive use among premenopausal women who are at risk of unintended pregnancy is important for the development of effective family planning policies. Previous studies have suggested that socioeconomic factors including age, education, residency, and occupation are among the main factors influencing contraceptive use [6,7,8,9,10,11]. Some studies have also reported that reproductive factors including marital age, gestational age, gravidity, parity, and birth interval also affect contraceptive use [7,8,9].

In addition, cultural and religious variables are known to influence the prevalence of contraceptive use [12,13]. Thus, identifying factors that influence the use of contraceptives, including OCs, in premenopausal women is essential for reducing the risk of unintended pregnancy. However, few studies have examined variables related to OC use in Korean women of reproductive age. Furthermore, few longitudinal epidemiological studies have identified trends in OC use in Korea.

The aim of the present study was to determine the prevalence and related factors of OC use in premenopausal Korean women using data from the Korea National Health and Nutrition Examination Survey (KNHANES), a nationally representative survey conducted in the Republic of Korea. We analyzed the factors associated with such use and changes in its prevalence from 2010 to 2019.

## 2. Methods

### 2.1. Study Population

We used secondary data from the 2010–2019 KNHANES, which were provided by the Korea Centers for Disease Control and Prevention. To obtain nationally representative samples of the Korean population, the KNHANES used a stratified, multistage, cluster-sampling design with proportional allocation based on the National Census Registry. This study included data of 14,386 premenopausal women aged 20–59 years, who were not pregnant, surveyed across 10 consecutive years. The study protocol was approved by the Korean Ministry of Health and Welfare, and the study was conducted in accordance with the Ethical Principles for Medical Research Involving Human Subjects defined by the Helsinki Declaration. All participants provided written informed consent.

### 2.2. Variable Definitions

The KNHANES employed well-established questions to determine participants’ demographic and socioeconomic characteristics, including age, gender, education level, income, smoking habit, alcohol consumption, and gravidity. Education level was categorized as less than a high school diploma, high school diploma, college, or advanced degree. Alcohol consumption was assessed by questions about drinking behavior during the month before the interview. Subjects were asked about the average frequency of alcoholic beverage intake. Alcohol consumption frequency was classified as none, drinking less or equal to once a month, and drinking more than once a month. Height and weight were measured while participants wore light clothing and no shoes. Body mass index (BMI) was calculated as weight (in kg) divided by the square of height (in m). Subjects were categorized as underweight (BMI < 18.5 kg/m^2^), normal weight (18.5 kg/m^2^ ≤ BMI < 23.0 kg/m^2^), overweight (23.0 kg/m^2^≤ BMI < 25.0 kg/m^2^), or obese (BMI ≥ 25.0 kg/m^2^) based on World Health Organization (WHO) definitions for Asian populations.

### 2.3. Statistical Analysis

We calculated frequencies (with percentages) for categorical variables and means and standard deviations (SDs) for continuous variables, when describing the sample population. We compared continuous variables between OC users and non-users using the *t*-test. The statistical significance of differences was determined using the Mantel–Haenszel chi-squared test. Sampling weights were used to determine the annual prevalence of OC use. The sampling weights were estimated by the following three factors: the sample selection probability, the adjustment for non-response, and the post-stratification factor [14]. Sampling weights calculated by the Korea Centers for Disease Control and Prevention were used in this study. The use of sample weights helped to account for unequal probability sampling among the different strata and ensured the representativeness of the survey results at the national level. The statistical analyses also took account of the survey design, and appropriate procedures in SAS (‘surveymeans’) were applied to the weighted data to calculate the mean prevalence (with a 95% confidence interval, CI) of OC use. All statistical analyses were conducted using SAS software (ver. 9.4; SAS Institute, Cary, NC, USA).

## 3. Results

A total of 14,386 women aged 20–59 years were included in this study; their demographic characteristics are presented in Table 1. The mean age and BMI of the study population were 37.2 years and 22.6 kg/m^2^, respectively.

**Figure 1:**
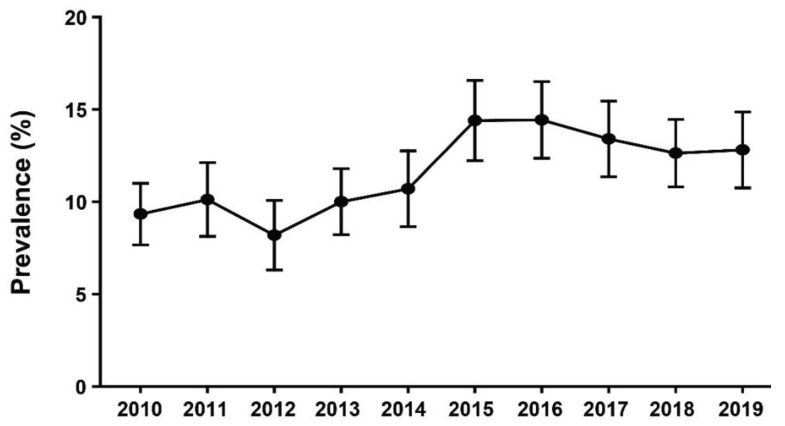
Annual prevalence (95% confidence interval) of OC use from 2010 to 2019.

Among Korean women aged 20–59 years, the estimated annual mean prevalence of OC use was 8.2–10.7% during 2010–2014. Compared to 2010–2014, this value increased to 12.6–14.4% during 2015–2019 (Figure 1).

**Figure 2:**
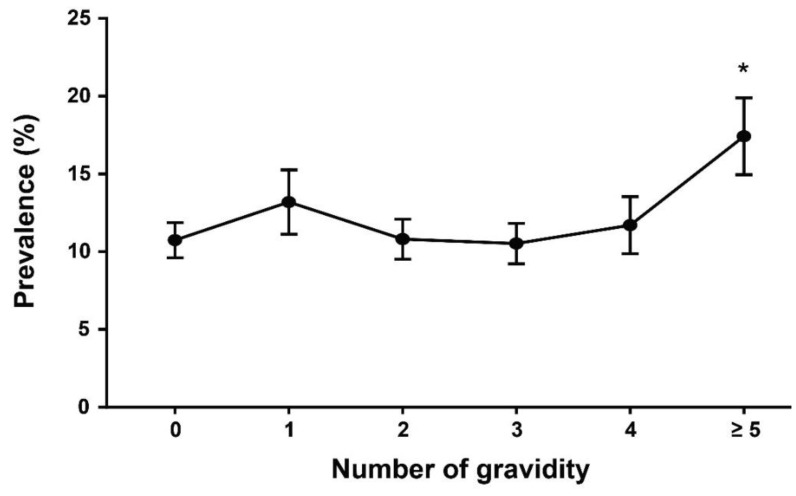
Annual prevalence (95% confidence interval) of OC use according to gravidity. * *p* < 0.01, compared with the other groups.

Figure 2 shows the prevalence of OC use across categories of gravidity. The prevalence of OC use was significantly higher among subjects with the higher (≥5) than lower gravidity (<5) (*p* < 0.01).

The participants’ basic characteristics are presented in Table 2 by OC use and the study outcomes. As the education level decreased, the participants’ use of OCs increased (*p* < 0.001). In addition, women belonging to the lowest income quartile were more likely to use OCs than those in the highest income quartile (*p* = 0.011). Cigarette smoking (*p* < 0.001) and alcohol drinking (*p* = 0.022) were associated with increased OC use. However, OC use was not significantly correlated with BMI, marital status, employment status, or area of residence.

## 4. Discussion

OCs containing estrogen and progestin have long been used as an effective contraceptive method. Contemporary third- or fourth-generation OCs, which have fewer side effects than earlier generations [15,16,17], are now widely used worldwide as a family planning method. In the present nationwide survey of premenopausal Korean women aged 20–59 years, the most recent (2019) annual prevalence rate of OC use was 12.8%, which is higher than that in Japan and lower than those in many European countries, such as the United Kingdom and France [3,18,19,20]. This variation in OC use could be attributed to variations in cultural or religious attitudes, as well as the accessibility and availability of OCs in each country.

In this study, the prevalence of OC use between 2010 and 2014 fluctuated between 8.2 and 10.7% but increased sharply after 2015. The increased use of OCs in Korea has two possible explanations. First, a national survey examining the use of contraceptive methods among Korean married women indicated that condoms were the most commonly used form of contraception in Korea. On the other hand, the use of intrauterine devices (IUDs) as a method of contraception in Korea has significantly decreased since 2015 [21], which may have contributed to the greater use of OCs in addition to condoms.

Disasters at the national level may also, at least in part, have influenced patterns of contraception use. In April 2014, a large ferry disaster caused severe distress in Korean society. Studies have provided evidence of the long-lasting negative psychological impact of this disaster, manifested as depressive symptoms and anxiety not only among the victims’ families and community residents, but also in the Korean population as a whole [22,23,24,25]. Previous studies have reported that social disasters affect contraceptive behavior, and that depressive symptoms may affect methods of contraception [26,27]. In addition, a recent study reported that social disasters significantly reduce the fertility rate for a fairly long period of time [28]. Thus, the notable increase in OC use in 2015 may be partly attributable to the adverse psychological effects of the ferry disaster.

In addition, the present study showed that gravidity affects OC use. Similar to the results of this study, a study analyzing US National Health and Nutrition Examination Survey (NHANES) data [29] identified differences in trends of OC use according to pregnancy history, suggesting that the use of OC to avoid unintended pregnancies may increase with higher gravidity.

The present study found that sociodemographic factors including education level, household income, smoking, and drinking affected OC use. Women with lower educational and socioeconomic status tend to have sex at a younger age [30]. Therefore, young women with low educational attainment and/or low household income may be more likely to require contraceptive methods, including OCs. Furthermore, smoking prevalence is known to be higher in socioeconomically vulnerable groups [31]. Additionally, similar to the results of the present study, previous studies indicated that women who reported using OCs were more likely to smoke and drink than non-OC users [32,33]. Thus, there may be a relationship between smoking/drinking and early sexual initiation, which could lead to increased OC use.

This study has some limitations. Given the complexity of factors influencing drug use behavior, it is reasonable to assume that there are additional factors or demographic variables that are not accounted for in our model. Factors such as associated comorbidities, use of contraindicated drugs, and cultural or religious variables need to be included in the analysis of future studies. Another limitation of this study is that self-reporting OC use and sociodemographic variables, including alcohol drinking and smoking, may lead to misclassification and recall bias. The results of this study suggest that, when formulating a family planning policy including the use of contraceptives, it is necessary to consider sociodemographic factors such as the target group’s education level, alcohol drinking and smoking habits as well as the number of pregnancies.

## 5. Conclusions

In this study, using nationally representative data, the prevalence of OC use between 2010 and 2019 ranged from 8.2 to 14.4%, with the prevalence increasing since 2015. OC use was related to gravidity, education level, household income, smoking, and drinking. The results of this study could be used to predict trends in contraception use and will be valuable for establishing policies to improve the availability and accessibility of OCs for specific groups.

## Figures and Tables

**Table 1:** Demographic characteristics of the participants.

Characteristics	*n*	%
Age (years)		
20–29	3255	22.6
30–39	4887	34.0
40–49	5172	36.0
50–59	1072	7.4
BMI		
<18.5	1204	8.4
18.5–22.9	7664	53.3
23–25	2421	16.8
>25	3097	21.5
Education		
<High School Diploma	891	6.2
High School	5827	40.5
High shool < – ≤ Undergraduate degree	6869	47.8
Advanced Degree	799	5.5
Average Household Income (USD/month)		
<1600	3446	23.9
1600–2299	3580	24.9
2300–3300	3678	25.6
>3300	3682	25.6
Cigarette Smoking		
Yes	1021	7.1
No	13,365	92.9
Alcohol Drinking		
Yes	8698	60.5
No	5688	39.5
Number of Gravidity		
0	3971	27.6
1–3	7487	52.0
>3	2928	20.4

**Table 2:** Sociodemographic factors affecting oral contraceptive (OC) use among married Korean women.

Characteristics	OC User (*n* = 1573)	Non-OC User (*n* = 12,813)	*p*-Value ^1^
Age (years), mean (SD)	37.4 (9.1)	37.2 (8.8)	0.386
BMI, mean (SD)	22.8 (3.8)	22.6 (3.7)	0.051
Marriage, *n* (% within group)			0.619
Yes	9513 (74.2)	1177 (74.8)	
No	3300 (25.8)	396 (25.2)	
Education, *n* (% within group)			<0.001
<High school diploma	184 (11.7)	707 (5.5)	
High school	668 (42.5)	5159 (40.3)	
High shool < – ≤ Undergraduate degree	654 (41.6)	6215 (48.5)	
Advanced degree	67 (4.2)	732 (5.7)	
Income, *n* (% within group)			0.011
<1600	413 (26.3)	3033 (23.7)	
1600–2299	409 (26.0)	3171 (24.7)	
2300–3300	364 (23.1)	3314 (25.9)	
>3300	387 (24.6)	3295 (25.7)	
Cigarette smoking, *n* (% within group)			<0.001
Yes	225 (14.3)	796 (6.2)	
No	1348 (85.7)	12,017 (93.8)	
Alcohol drinking, *n* (% within group)			<0.001
Never	239 (15.2)	2636 (20.6)	
≤1 drink/month	594 (37.8)	5088 (39.7)	
>1 drink/month	740 (47.0)	5089 (39.7)	
Employment, *n* (% within group)			0.055
Yes	979 (62.2)	7652 (59.7)	
No	594 (37.8)	5161 (40.3)	
Residence, *n* (% within group)			0.498
Urban	1361 (86.5)	11,164 (87.1)	
Rural	212 (13.5)	1649 (12.9)	

^1^ *p*-values were determined using the *t*-test or Mantel–Haenszel chi-square test, based on comparison between the OC and non-OC groups.

## Data Availability

The data used to support the findings of this study are available from the corresponding author upon request.
